# Rigid-foldable cylindrical origami with tunable mechanical behaviors

**DOI:** 10.1038/s41598-023-50353-4

**Published:** 2024-01-02

**Authors:** Fengrui Liu, Tatsuro Terakawa, Siying Long, Masaharu Komori

**Affiliations:** Department of Mechanical Engineering and Science, Kyoto University, Kyoto daigaku-katsura, Nishikyo-ku, Kyoto, 615-8540 Japan

**Keywords:** Mechanical engineering, Mechanical properties

## Abstract

Rigid-foldable origami shows significant promise in advanced engineering applications including deployable structures, aerospace engineering, and robotics. It undergoes deformation solely at the creases during the folding process while maintaining rigidity throughout all facets. However, most types of cylindrical origami, such as Kresling origami, water-bomb origami, and twisted tower origami, lack rigid-foldability. Although shape transformation can be achieved through elastic folding, their limited rigid foldability constrains their engineering applications. To address this limitation, we proposed a type of cylindrical origami inspired by Kresling origami, named foldable prism origami (FP-ori), in this paper. FP-ori possesses not only rigid-foldability but also several tunable properties, including flat-foldability, self-locking, and bistability. The geometric properties of FP-ori were analyzed and the relationship between different parameters and tunable mechanical behaviors were verified through finite element method simulations, as well as experiments using paper models. Furthermore, we proposed stacked structures composed of multiple cubic FP-ori units, the rotation directions of which could be controlled through the combination arrangement. And drawing inspiration from kirigami, a negative Poisson’s ratio tessellation structure was created. These results indicated that FP-ori has substantial potential for broad application in engineering fields.

## Introduction

Origami is the art of transforming a two-dimensional (2D) sheet of paper into a well-designed three-dimensional (3D) configuration. Origami’s foldability has led to its wide-ranging applications in engineering, spanning diverse fields including metamaterials^1^, aerospace^2^^,^^3^, medical devices^4^^,^^5^, robotics^6^^,^^7^, and energy absorption^8^.

Unlike 2D origami, cylindrical origami has some creases that must be glued together to form a 3D unfolded configuration, which is more suitable for deployable structures and energy absorbers^9^. Various types of cylindrical origami exist, such as Kresling origami, twisted tower origami, Yoshimura origami, and water-bomb origami. Kresling origami usually finds application in crawling robots^10^ and reconfigurable antennas^11^ because of its bistability. Twisted tower origami offers multiple degrees of freedom in motion, including extension, contraction, and bending^12^. Inspired by this origami, a flexible gripper was designed for grasping objects with diverse shapes^13^. Yoshimura origami, which utilizes multiple identical triangles, exhibits superior energy absorption capacity than that of a homogeneous tube during compression^14^. Water-bomb origami can be used to fix cylindrical and axisymmetric curved surfaces, showing potential in deployable structures^15^^,^^16^. Moreover, additional processing can be applied to conventional origami configurations to enhance their functions and applications. For instance, Zhang et al. proposed a structure with programmable multi-stability, achieved by combining foldable kirigami cuboids with elastic hinges^17^. Filipov assembled various Miura origami tubes to create a metamaterial that can be deployed, stiffened, and tuned^18^. A radially closable structure (RC-ori), a modified form of Kresling origami with crease lines, has also been proposed^19^. Adding incisions on the creases of Kresling origami significantly reduced the bistable actuation force during compression^20^. Cylindrical origami holds significant potential for a wide range of applications in deployable structures, origami robots, and metamaterials.

In the theory and application of origami, two crucial properties are considered: rigid-foldability and flat-foldability. Origami structures with rigid-foldability enable facets to rotate solely around the creases without undergoing deformation throughout the folding process^21^ and origami structures with flat-foldability can be folded into a flat overlapped sheet^22^. Rigid-foldability ensures smooth folding and allows for the structure to maintain its shape when folded, which is essential for designing rigid-material origami robots. Flat-foldability permits efficient storage and transportation of the deployable structure^23^. However, most cylindrical origami structures lack these two properties, limiting their application range to soft materials or elastic hinges. To address this limitation, Miura and Tachi proposed a new origami pattern named Tachi-Miura origami, which is rigid-foldable and cylindrical. It is formed by connecting differently designed Miura origami units to form a retractable cylindrical structure^24^. Chen proposed a method for constructing an extended family of rigid-foldable origami tubes^25^. Some researchers have also focused on folding a box-like carton into a flat configuration^26^. Wu proposed a solution for folding tall, rigid shopping bags without a top facet^27^ and Gu created an origami cube that is rigid and flat-foldable with one degree of freedom^28^. Nevertheless, in existing cylindrical origami types there is not a configuration that possesses rigid-foldability, flat-foldability, and a regular prismatic shape, simultaneously. Hence, inspired by Kresling origami, we introduced a new cylindrical origami, named foldable prism origami (FP-ori), with diagonal cuts on the side facets in this paper. We calculated the relationship between the parameters and examined the effect of parameter values on the mechanical behaviors. Additionally, 3D multi-unit stacked structures are proposed for further exploration.

## Results

### Foldable prism origami

**Figure 1:**
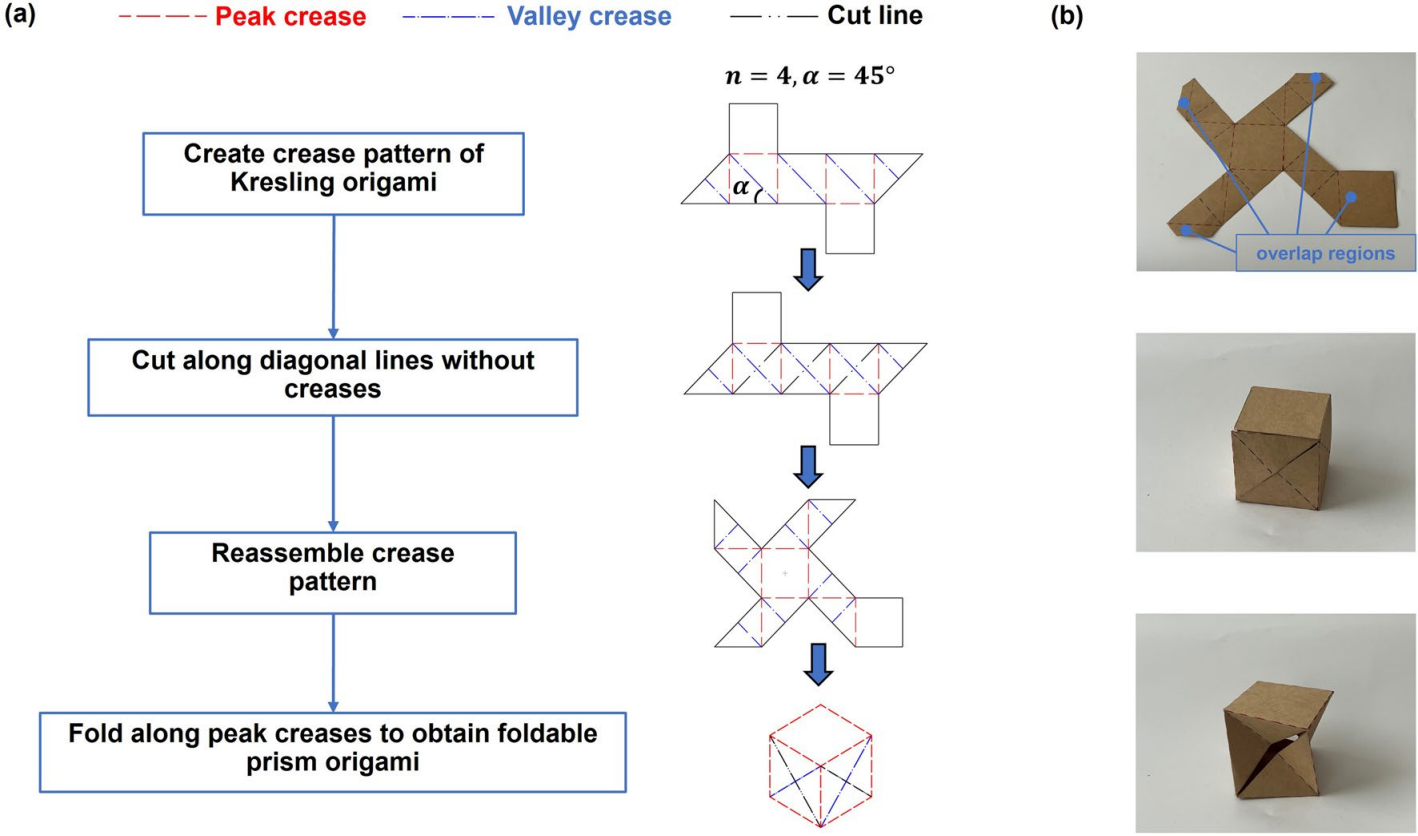
Transformation process of FP-ori. (**a**) Transformation from Kresling origami to FP-ori ($n=4$ and $\alpha =45^\circ$). (**b**) Fabrication process from crease pattern to 3D FP-ori structure.

FP-ori drew inspiration from Kresling origami and the transformation from it to FP-ori is illustrated in Fig. 1a. A foldable cube with $n=4$, where $n$ is the number of polygon sides, is used as an example to explain the transformation process. The peak creases are depicted as red dashed line, valley creases as blue dashed-dotted line, and cut lines as black dashed double-dotted line. The crease pattern of a Kresling origami prism consists of several right triangles, and we add cut lines along the diagonal without creases. Subsequently, the crease pattern is reassembled into a windmill-like shape. Figure 1b demonstrates the final step, how to transform the crease pattern into a 3D FP-ori structure. During the fabrication process, it is necessary to fold along creases and connect the overlapping regions using glue (the video of the fabrication process can be found in Movie S1). To provide a more intuitive demonstration of the transformation process of FP-ori, a method for directly converting a prism into FP-ori is proposed. Based on the final step, we can consider all edges of the cube as peak creases and add valley creases and cuts on the side facets. Taking one side facet as an example, if the valley crease goes from the lower-left vertex to the upper-right vertex, then the cut extends from the upper-left vertex to the lower-right vertex. The directions of valley creases and cuts are identical on all side facets. This transformation method is referred to as the FP-ori method. In addition to square prisms, the FP-ori method can be used to rigidly fold all the polygonal prisms. (Please refer to Supplementary Material, Sect. 1, for the crease patterns and flat configurations of three types of FP-ori: a triangular prism, a pentagonal prism, and a hexagonal prism).

### Geometry model

**Figure 2:**
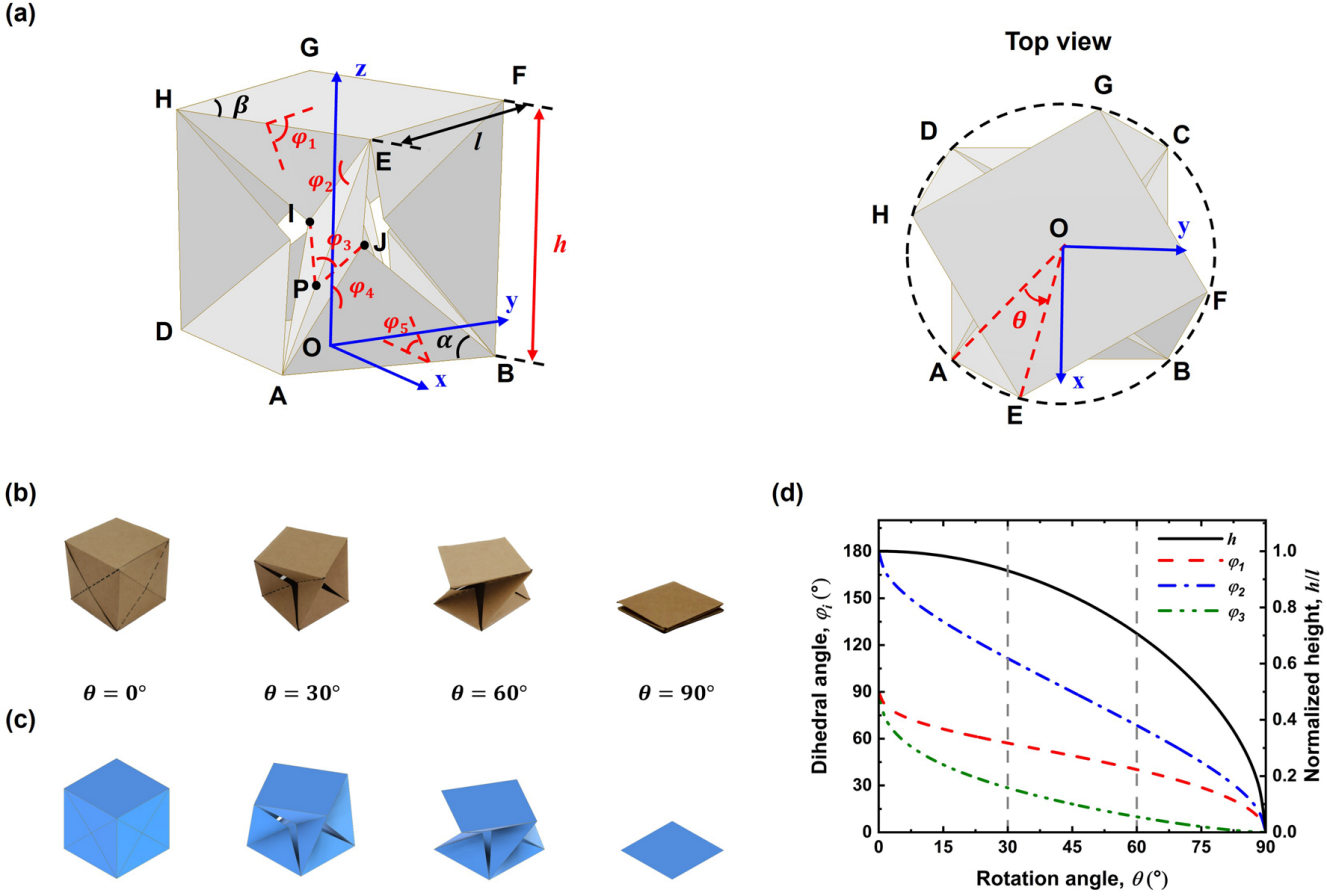
Geometric relationships of FP-ori. (**a**) Schematic diagram and top view of FP-ori with $n=4$ and $\alpha =45^\circ$. (**b**) Actual folding process and (**c**) simulation results. (**d**) Variation of ${\varphi }_{i}$ and $h$ during the folding process of FP-ori with $n=4$ and $\alpha =45^\circ$.

This section focuses on constructing the geometric model of FP-ori. An FP-ori model (i.e. $n=4$) with eight vertices, denoted as A to H, is illustrated in Fig. 2a as an example. The bottom facet (i.e. ABCD) and the top facet (i.e. EFGH) are two identical regular polygons with the same side length $l$, interior angle $\beta$, and the number of polygon sides $n$. Points I and J represent two intersection points of the diagonals. A Cartesian coordinate system with its origin at the center point O of the bottom facet (O-$xyz$) is established. The $x$-axis runs parallel to the vector $\overrightarrow{{\text{DA}}}$ and the $z$-axis is perpendicular to the bottom facet ABCD.

According to origami theory, we considered the facets as rigid panels and creases as axial rotation hinges. For the sake of convenience, we assumed that the top and bottom facets remain parallel to each other during the entire folding process, ensuring geometric symmetry in the four limbs (ABEH, BCFE, CDGF, and DAHG). This assumption results in a single degree of freedom for the entire folding process, establishing a one-to-one correspondence for all parameters. The coordinates of points A, B, E, I, and J are provided in Table 1. Here, $\alpha$ represents the angle between the diagonal BJ and the bottom side BA, where $0<\alpha \le \beta /2$, ${\varphi }_{i}$ ($i$=1, 2, 3, 4, 5) are the dihedral angles from the top facet to the bottom facet, as depicted in Fig. 2a, where $0<{\varphi }_{1}, {\varphi }_{3},{\varphi }_{5}\le \pi /2$ and $0<{\varphi }_{2},{\varphi }_{4}\le \pi$. Additionally, $h$ is the height from the top facet to the bottom facet, and $\theta$ signifies the rotation angle between the top and bottom facets.

**Table 1:** Coordinates of points A, B, E, I, and J.

Point	*x*-coordinate	*y*-coordinate	*z*-coordinate
A	${\text{tan}}(\beta /2)l/2$	$-l/2$	0
B	${\text{tan}}(\beta /2)l/2$	$l/2$	0
E	$\left[{\text{tan}}(\beta /2){\text{cos}}\theta +{\text{sin}}\theta \right.]l/2$	$\left[{\text{tan}}(\beta /2){\text{sin}}\theta -{\text{cos}}\theta \right.]l/2$	$h$
I	$[{\text{tan}}(\beta /2)-{\text{cos}}{\varphi }_{1}{\text{tan}}\alpha ]{\text{cos}}\theta l/2$	$[{\text{tan}}(\beta /2)-{\text{cos}}{\varphi }_{1}{\text{tan}}\alpha ]{\text{sin}}\theta l/2$	$h-{\text{sin}}{\varphi }_{1}{\text{tan}}\alpha l/2$
J	$[{\text{tan}}(\beta /2)-{\text{cos}}{\varphi }_{1}\cdot {\text{tan}}\alpha ]l/2$	0	$l{\text{sin}}{\varphi }_{1}{\text{tan}}\alpha /2$

Because the deformation of the surface is not considered, the distance between points E and A and that between points E and J are calculated as follows:

1 $$\left|\overrightarrow{{\text{EA}}}\right|=l{\text{tan}}\alpha ,$$

2 $$\left|\overrightarrow{{\text{EJ}}}\right|=l/(2{\text{cos}}\alpha ),$$

From the definition of the dihedral angle, the following equation can be obtained:

3 $${\text{cos}}{\varphi }_{4}={\overrightarrow{N}}_{ABJ}\cdot {\overrightarrow{N}}_{AEJ},$$

where ${\overrightarrow{N}}_{ABJ}$ and ${\overrightarrow{N}}_{AEJ}$ are the unit normal vectors of facets ABJ and AEJ, respectively.

Point P is the midpoint of line AE. Therefore, line segments IP and JP are respectively perpendicular to line AE. In the triangle PIJ, according to the law of cosines, the following equation can be obtained:

4 $${\left|\overrightarrow{{\text{IJ}}}\right|}^{2}={\left|\overrightarrow{{\text{PI}}}\right|}^{2}+{\left|\overrightarrow{{\text{PJ}}}\right|}^{2}-2\left|\overrightarrow{{\text{PI}}}\right|\left|\overrightarrow{{\text{PJ}}}\right|{\text{cos}}{\varphi }_{3}.$$

Some parameters are identical throughout the folding process because of symmetry; they can be written as:

5 $$\left\{\begin{array}{c}{\varphi }_{1}={\varphi }_{5}\\ {\varphi }_{2}={\varphi }_{4}\end{array}.\right.$$

Solving and arranging Eqs. (1), (2), (3), and (4) yields the following equations:

6 $$h=l\cdot \sqrt{{{\text{tan}}}^{2}\alpha -\frac{1-{\text{cos}}\theta }{2{{\text{cos}}}^{2}\left(\beta /2\right)},}$$

7 $${\left({\text{tan}}\frac{\beta }{2}\left({\text{cos}}\theta -1\right)+{\text{sin}}\theta +{\text{tan}}\alpha {\text{cos}}{\varphi }_{1}\right)}^{2}+\left({\text{cos}}\theta -{\text{sin}}\theta {{\text{tan}}}^{2}\frac{\beta }{2}+{\left(\frac{2h}{l}-{\text{tan}}\alpha {\text{sin}}{\varphi }_{1}\right)}^{2}\right){{\text{cos}}}^{2}\alpha =1,$$

8 $${\text{cos}}{\varphi }_{2}=\frac{2{{\text{sin}}}^{4}\alpha +1-3{{\text{sin}}}^{2}\alpha }{2{{\text{sin}}}^{2}\alpha (1-{{\text{sin}}}^{2}\alpha )}-\frac{{\text{cos}}\theta +{\text{cos}}\left(\theta +\beta \right)}{4\left(1-{{\text{sin}}}^{2}\alpha \right){{\text{cos}}}^{2}\beta /2{{\text{tan}}}^{2}\alpha },$$

9 $${\text{cos}}{\varphi }_{3}=1-\frac{{4\cdot {\text{cos}}}^{2}(\beta /2)\cdot {{\text{tan}}}^{2}\alpha \cdot {\left(h/l-{\text{tan}}\alpha \cdot {\text{sin}}{\varphi }_{1}\right)}^{2}}{1-{\text{cos}}\theta }.$$

Therefore, ${\varphi }_{i}$ and $h$ can be obtained from Eqs. (6), (7), (8), and (9) for various values of $\alpha$, $\beta$, and $\theta$. $\beta$ is determined from the number of polygon sides $n$ as:

10 $$\beta =\frac{n-2}{n}\uppi .$$

Figure 2b illustrates the folding process of a paper-made cubic FP-ori in four stages corresponding to $\theta =0^\circ$, $30^\circ$, $60^\circ$, and $90^\circ$, and Fig. 2c displays the simulation results obtained using Autodesk Inventor Professional 2021. Based on the previously described geometry model, the variation of ${\varphi }_{i}$ during the folding process of this FP-ori was calculated and the results are illustrated in Fig. 2d. It is evident that there is a one-to-one correspondence between ${\varphi }_{i}$ and $\theta$, which verifies FP-ori as a type of rigid-foldable origami. In simpler terms, this FP-ori can be rigidly folded from a cube into a square resulting in a significant volume change, rendering it suitable for using in deployable structures.

### Mechanical behaviors of FP-ori with different parameters

In the case of FP-ori, the origami configuration is completely determined by three main geometric parameters, namely $l$, $n$, and $\alpha$, while *l* controls the structure proportionally scaled up and down. Consequently, an FP-ori configuration can be predominantly determined by $n$ and $\alpha$ This section mainly delves into the mechanical behaviors of FP-ori with different values of $n$ and $\alpha$.

#### Flat foldability

**Figure 3:**
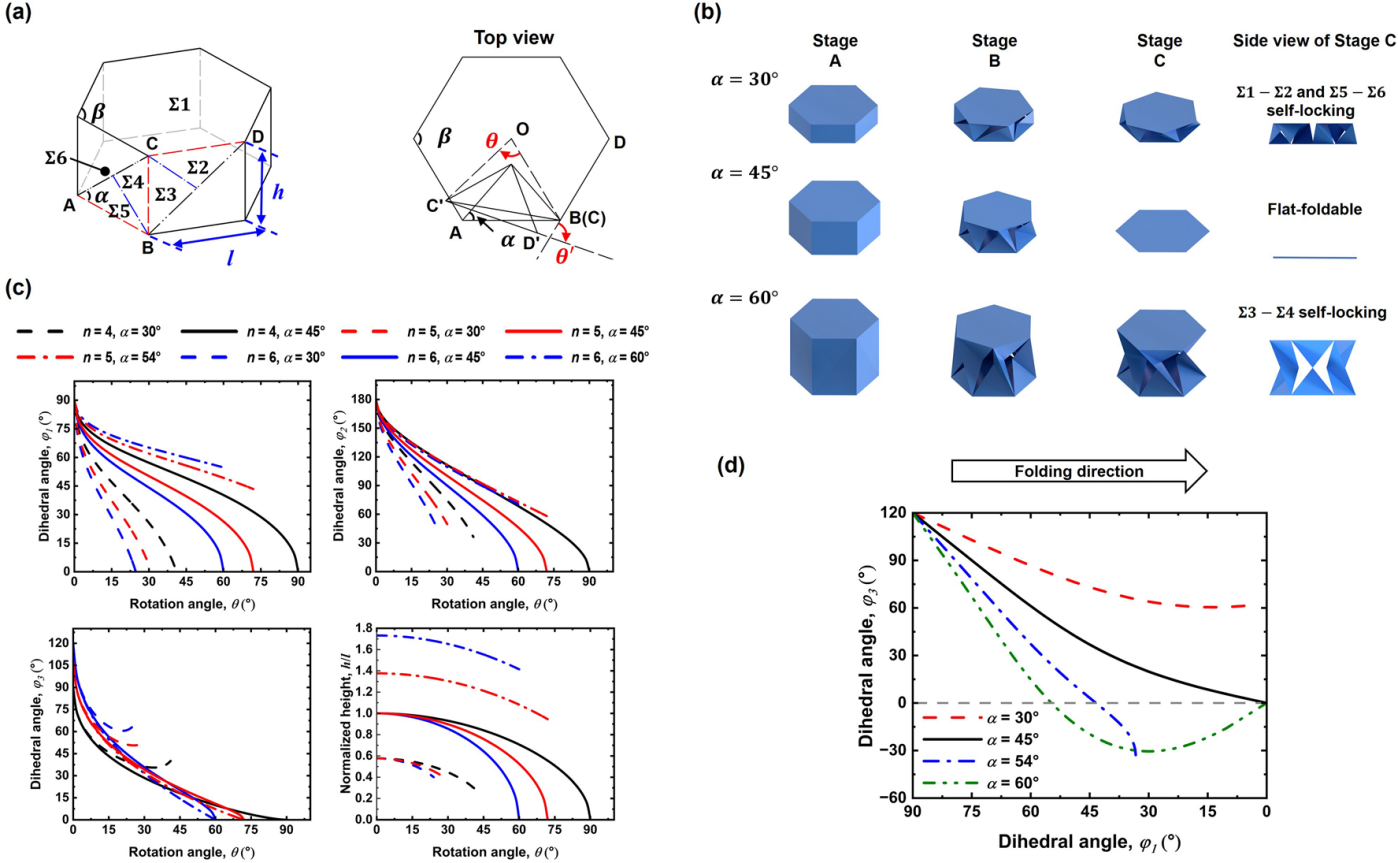
Mechanical behaviors of FP-ori with various parameter values. (**a**) Schematic diagram and top view of a single limb (ABCD) in hexagonal FP-ori (**b**) Folding process of FP-ori structure with various values of $\alpha$ ($\alpha =30^\circ$, $45^\circ$, $60^\circ$). (**c**) Variation of ${\varphi }_{1}$, ${\varphi }_{2}$, ${\varphi }_{3}$, and $h/l$ with $n$ and $\alpha$. (**d**) Variation of ${\varphi }_{1}$ and ${\varphi }_{3}$ with $\alpha$ ($\alpha =30^\circ$, $45^\circ$, $54^\circ$, $60^\circ$).

The preceding calculations and simulations demonstrated that FP-ori with $n=4$ and $\alpha =45^\circ$ can transform from a 3D prism to a 2D polygon plane after rigid folding, indicating its flat-foldability. The hexagonal prism ($n=6$), as shown in Fig. 3a, is utilized to determine the condition for flat-foldability. The top and bottom facets are designated as $\Sigma 1$ and $\Sigma 6$, respectively, while the triangular facets are denoted as $\Sigma 2$, $\Sigma 3$, $\Sigma 4$, and $\Sigma 5$, as depicted in Fig. 3a. The top view in Fig. 3a reveals that only one of the limbs becomes flat without considering the influence of facets $\Sigma 1$ and $\Sigma 6$. Rotation angle $\theta$ represents the rotation angle of point ${\text{C}}$ around center point ${\text{O}}$ of the bottom facet and the rotation angle ${\theta }^{\mathrm{^{\prime}}}$ signifies the angle between line ${\text{CD}}$ and line ${{\text{C}}}^{\mathrm{^{\prime}}}{{\text{D}}}^{\mathrm{^{\prime}}}$, where ${{\text{C}}}^{\mathrm{^{\prime}}}{{\text{D}}}^{\mathrm{^{\prime}}}$ indicated the position of line ${\text{CD}}$ after folding. For this origami structure to be flat-foldable, the top facet should remain a hexagon and the center points of the top and bottom facets should coincide during the folding process. Consequently, the two rotation angles $\theta$ and ${\theta }^{\mathrm{^{\prime}}}$ must be identical, yielding the following equations:

11 $${\left|\overrightarrow{\mathrm{C}^{\mathrm{^{\prime}}}{\text{B}}}\right|}^{2}={\left|\overrightarrow{{{\text{C}}}^{\mathrm{^{\prime}}}{\text{O}}}\right|}^{2}+{\left|\overrightarrow{{\text{BO}}}\right|}^{2}-2\left|\overrightarrow{{{\text{C}}}^{\mathrm{^{\prime}}}{\text{O}}}\right|\left|\overrightarrow{{\text{BO}}}\right|{\text{cos}}\theta ,$$

12 $${\theta }^{\mathrm{^{\prime}}}=4\alpha -\beta .$$

Substituting Eq. (11) into Eq. (12), the relationship equation can be rewritten as:

13 $$\left({\text{tan}}\alpha -{\text{tan}}\left(\frac{\pi \left(n-2\right)}{2n}\right)\right){\text{cos}}2\alpha =0.$$

Solving Eq. (13) yields the following condition for the flat-foldability of FP-ori:

14 $$\alpha =\frac{\pi (n-2)}{2n}=\frac{\beta }{2}\;\mathrm{or}\;\alpha =\frac{\uppi }{4}.$$

FP-ori can be folded into a plane without facet or crease deformation only when $\alpha =\beta /2$ or $\alpha =\pi /4$.

#### Self-locking

Figure 3b displays three types of hexagonal FP-ori, with $\alpha =30^\circ$, $45^\circ$, and $60^\circ$, respectively. The folding process is segmented into Stages A, B, and C, representing the initial configuration, intermediate configuration, and final configuration of FP-ori, respectively. When the hexagonal FP-ori with $\alpha =30^\circ$ reaches the folding limit at Stage C, facets $\Sigma 1$ and $\Sigma 2$ as well as facets $\Sigma 5$ and $\Sigma 6$ interfere with each other and there remains a certain angle between facets $\Sigma 3$ and $\Sigma 4$. This configuration is referred to as $\Sigma 1$-$\Sigma 2$ and $\Sigma 5$-$\Sigma 6$ self-locking. When $\alpha =45^\circ$, all facets align simultaneously within the same plane at Stage C, which demonstrates the aforementioned rigid and flat-foldability of FP-ori. For FP-ori with $\alpha =60^\circ$, which also satisfies the flat-foldability condition, facets $\Sigma 3$ and $\Sigma 4$ yet interfere with each other, characterizing it as $\Sigma 3$-$\Sigma 4$ self-locking.

To clarify the self-locking conditions of FP-ori, the variations of ${\varphi }_{1}$, ${\varphi }_{2}$, ${\varphi }_{3}$, and $h/l$ of FP-ori with $n$ and $\alpha$ are illustrated in Fig. 3c. The solid lines in the figure indicate that ${\varphi }_{i} (i=1, 2, 3)$ and $h/l$ become zero simultaneously at the end of the folding process only when $\alpha =45^\circ$. For cases involving $n=5$ and $\alpha =54^\circ$ or $n=6$ and $\alpha =60^\circ$, represented by the dashed-dotted lines, ${\varphi }_{3}$ is zero while ${\varphi }_{1}$ and ${\varphi }_{2}$ are not zero at the end of the folding process, even though the flat-foldability condition is satisfied, which is attributed to $\Sigma 3$-$\Sigma 4$ self-locking. When $\alpha =30^\circ$, as indicated by the dashed lines, only ${\varphi }_{1}$ becomes zero at the end of the folding process, signifying $\Sigma 1$-$\Sigma 2$ self-locking. Consequently, FP-ori can exhibit rigid and flat-foldable only when $\alpha =45^\circ$; self-locking occurs when $\alpha \ne 45^\circ$. It is apparent that $\Sigma 1$-$\Sigma 2$ and $\Sigma 5$-$\Sigma 6$ self-locking arises when $\alpha <45^\circ$ and $\Sigma 3$-$\Sigma 4$ self-locking occurs when $\alpha >45^\circ$. These conclusions are consistent with the results obtained from the aforementioned simulation.

#### Bistability

Kresling origami structures with specific parameters exhibit two unstrained configurations during the folding process. Building upon the calculations in the previous section, it has been observed that certain FP-ori structures cannot be folded flat even when they satisfy the flat-foldability condition. This implies that some FP-ori structures may also possess two unstrained configurations. As a result, it is speculated that after reaching the self-locking state, FP-ori under certain parameters may exhibit bistability, similar to that of Kresling origami.

For instance, self-locking structures with $\alpha =\beta /2$ (e.g. $n=5$ and $\alpha =54^\circ$ or $n=6$ and $\alpha =60^\circ$) satisfy the flat-foldability condition, as previously discussed. The structure should undergo no facet deformation in these two configurations (self-locking and flat-folded states). Put differently, no deformation energy is generated in these two states, whereas deformation energy exists between these states. To validate this, it is assumed that facets Σ3 and Σ4 can penetrate each other after self-locking, causing ${\varphi }_{3}$ to negative. The negative value is used to reflect the degree of deformation, with a larger absolute value of the angle ${\varphi }_{3}$ indicating a greater stored energy for the entire structure.

Figure 3d illustrates the variations of ${\varphi }_{1}$ and ${\varphi }_{3}$ during the folding process of hexagonal FP-ori ($n=6$) with $\alpha =30^\circ$, $45^\circ$, $54^\circ$, and $60^\circ$. The gray dotted line serves as the positive and negative dividing line for ${\varphi }_{3}$. The figure shows that the structure is both rigid and flat-foldable when $\alpha =45^\circ$ because ${\varphi }_{1}$ and ${\varphi }_{3}$ simultaneously reach zero. Self-locking occurs at $\alpha =30^\circ$, $54^\circ$, and $60^\circ$. When $\alpha =30^\circ$, only ${\varphi }_{1}$ becomes zero, indicating that $\Sigma 1$-$\Sigma 2$ self-locking occurs but $\Sigma 3$ and $\Sigma 4$ do not interfere with each other. For $\alpha =54^\circ$ and $60^\circ$, ${\varphi }_{3}$ becomes zero first, signifying $\Sigma 3$-$\Sigma 4$ self-locking, while the trend of the curves in these two configurations is distinct thereafter. When $\alpha =54^\circ$, ${\varphi }_{3}$ does not return to zero with an increase in ${\varphi }_{1}$, but when $\alpha =60^\circ$, ${\varphi }_{3}$ crosses the zero line and eventually returns to zero. Consequently, it is speculated that FP-ori with $\alpha =60^\circ$ possesses bistability because ${\varphi }_{3}$ has two zero points during the folding process, whereas FP-ori with $\alpha =54^\circ$ should be a monostable structure as ${\varphi }_{3}$ steadily decreases with only one zero point.

### Verification through finite element method simulation and experiment using paper model

**Figure 4:**
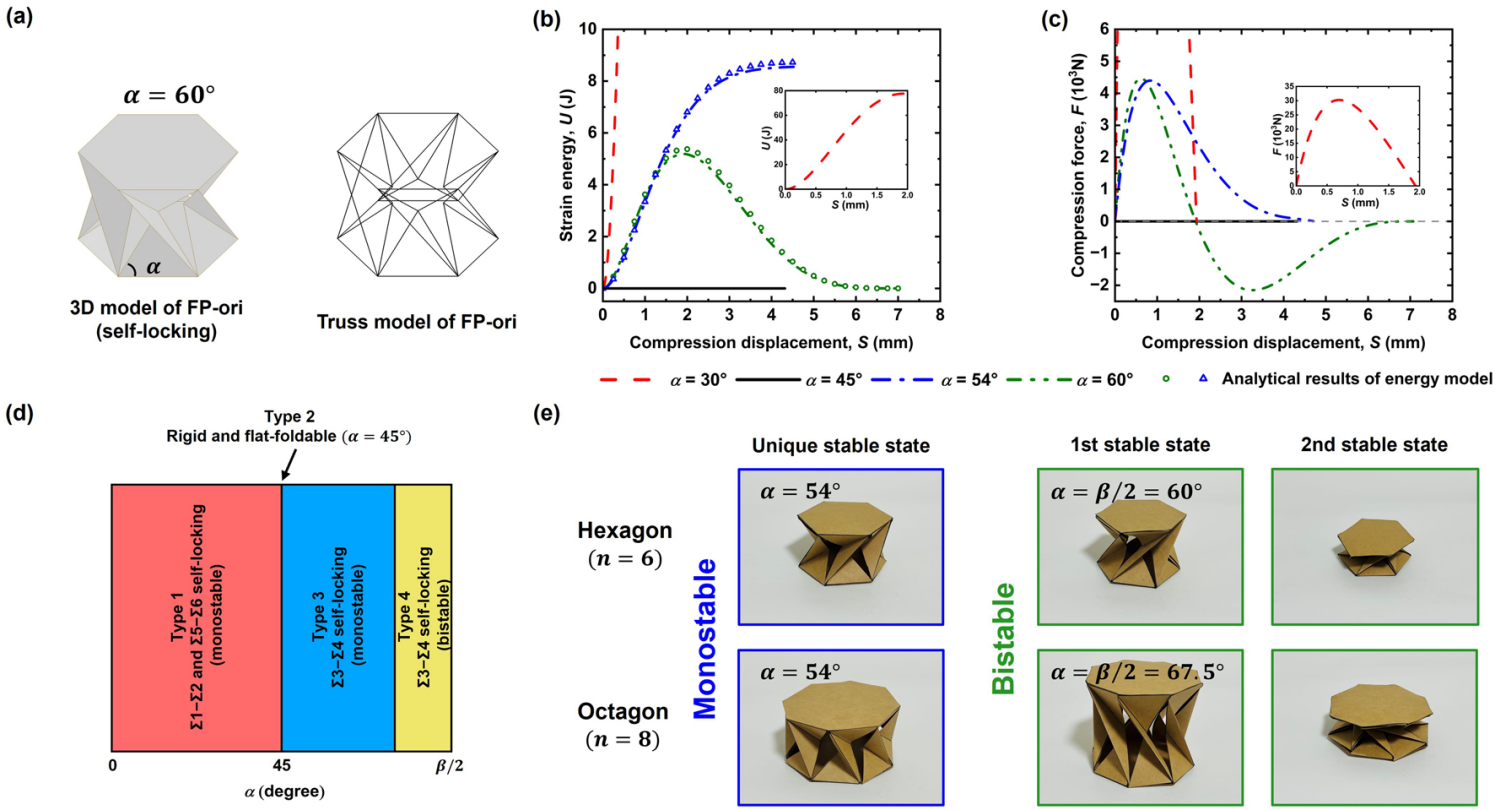
Results of FEM simulation and experiments using paper model. (**a**) Transformation from the cylindrical structure based on FP-ori with $\alpha =60^\circ$ to truss model. (**b**) Generated strain energy during the folding process for various $\alpha$ values. (**c**) Force–displacement curves for various $\alpha$ values. (**d**) Tunable mechanical behaviors with respect to the angle $\alpha$. (**e**) Comparative experiments between paper-made bistable FP-ori ($n=6, \alpha =60^\circ ;$ $n=8, \alpha =67.5^\circ$) and monostable FP-ori ($n=6, \alpha =54^\circ ;$ $n=8, \alpha =54^\circ$).

To verify the properties of FP-ori as discussed in the previous section, finite element method (FEM) simulations were conducted using Abaqus 2021. To simplify the simulation of FP-ori, as shown in Fig. 4a, a truss mode was established wherein all the creases were regarded as trusses capable of stretching and compressing without bending. The joints of the trusses were considered as hinges that could freely rotate in any direction. Young’s modulus was set at 210 MPa and Poisson’s ratio was 0.3. Each truss had a single element to prevent bending with the section area of 1 mm^2^ and the length of each hexagon side was 5 mm. Simulations were conducted on four FP-ori truss models with $\alpha =30^\circ$, $45^\circ$, $54^\circ$, and $60^\circ$ to verify the mechanical behaviors discussed in the previous section. The initial configuration of each FP-ori structure corresponds to the configuration where self-locking occurs when $\alpha =30^\circ$, $54^\circ$, and $60^\circ$. Since no self-locking occurs when $\alpha =45^\circ$, the initial position represents the configuration where ${\varphi }_{1}=45^\circ$(The compression process animations of these four FP-ori structures display in Movie S2, and the analysis can be found in Supplementary Material, Sect. 2).

The simulation results are presented in Fig. 4b. The strain energy $U$ represents the deformation energy in the folding process. The strain energy remains zero when $\alpha =45^\circ$, which verifies that FP-ori with $\alpha =45^\circ$ is a rigid and flat-foldable structure. When $\alpha =30^\circ$ or $54^\circ$, there is only one zero-energy point, namely in the initial configuration. The FP-ori structure with $\alpha =30^\circ$ can store significantly more strain energy than that with $\alpha =54^\circ$, indicating that FP-ori with $\alpha =30^\circ$ possesses a greater stiffness than that of FP-ori with $\alpha =54^\circ$. For $\alpha =60^\circ$, two zero-energy points are identified, consistent with bistability. Figure 4c illustrates the compression force–displacement curve, further confirming the validity of the previously drawn conclusions.

Furthermore, we established an energy model to elucidate the tunable mechanical behavior of FP-ori origami when $\alpha >45^\circ$(Please refer to Supplementary Material, Sect. 3, for calculation details). This model is also based on the origami truss model. The trusses composing the top and bottom facets are assumed to remain rigid throughout the entire process, while the axial trusses located between the top and bottom facets are considered to generate linear elastic deformation with the same Young’s module and section as in FEM. By summing the strain energy produced by each elastic truss during the compression process, we obtained the deformation energy of the entire FP-ori structure. By employing the principle of minimum total potential energy approach and utilizing MATLAB to solve partial differential equations, we obtained the analysis results depicted in Fig. 4b. It is evident that the results of the energy model are in good agreement with the FEM simulation results.

Therefore, we can summarize FP-ori’s tunable mechanical behaviors as follows (illustrated in Fig. 4d): when the angle $\alpha$ is between 0° and 45°, it belongs to type 1, $\Sigma 1$-$\Sigma 2$ and $\Sigma 5$-$\Sigma 6$ self-locking. At 45°, it exhibits rigid foldability and flat-foldability, referred to as type 2. Between 45° and $\beta /2$, it belongs to $\Sigma 3$-$\Sigma 4$ self-locking, and there exists a certain value that separates it into type 3, comprising a solely monostable state, and type 4, comprising a bistable state.

To further validate the previously obtained conclusions, we conducted two sets of comparative experiments involving monostable and bistable configurations. As shown in Fig. 4e, four models using paper were crafted with a thickness of 0.35 mm, including two hexagonal FP-oris ($n=6$) with the angle $\alpha$ of 54° and 60°, as well as two octagonal FP-oris ($n=8$) with the angle $\alpha$ of 54° and 67.5°, and all the polygons possess a side length of 40 mm. Notably, 60° ($n=6$) and 67.5° ($n=$ 8) are both equal to $\beta /2$. According to the previous conclusion, FP-ori should exhibit bistable behavior in these two cases. When we subjected these four structures to simple compression experiments, we found that for $n=6$ and $\alpha =54^\circ$, and $n=6$ and $\alpha =54^\circ$, only a unique stable state was observed. However, for $n=6$ and $\alpha =60^\circ$, and $n=8$ and $\alpha =67.5^\circ$, two clear bistable states were observed (The experimental process illustrated in Movie S3). Consequently, even when the paper thickness couldn't be negligibly small, we still obtained results consistent with FEM, confirming that FP-ori indeed possesses tunable mechanical behaviors under different parameters.

### Programmable multi-unit FP-ori structure

In the previous sections, we described the geometry, kinematics, and mechanical behaviors of an FP-ori unit. However, when multiple origami units are combined to form a complex structure, a wide range of properties can be achieved. Taking the Miura origami, the most widely recognized and extensively researched origami structure, as an example, when units with different parameters are combined, it exhibits the ability to design a ‘globally planar’ or ‘globally curved pattern’^29–33^. It also demonstrates properties such as self-locking^21^, graded stiffness^34^, and other interesting geometric and mechanical characteristics. Therefore, researching how to combine multiple origami units into complex origami structures holds profound significance for future research. In this section, we introduce methods for the vertically and horizontally connecting multiple FP-ori units, which can be employed to control the rotation direction and construct 3D structures with a negative Poisson’s ratio.

#### Vertically stacked structure

**Figure 5:**
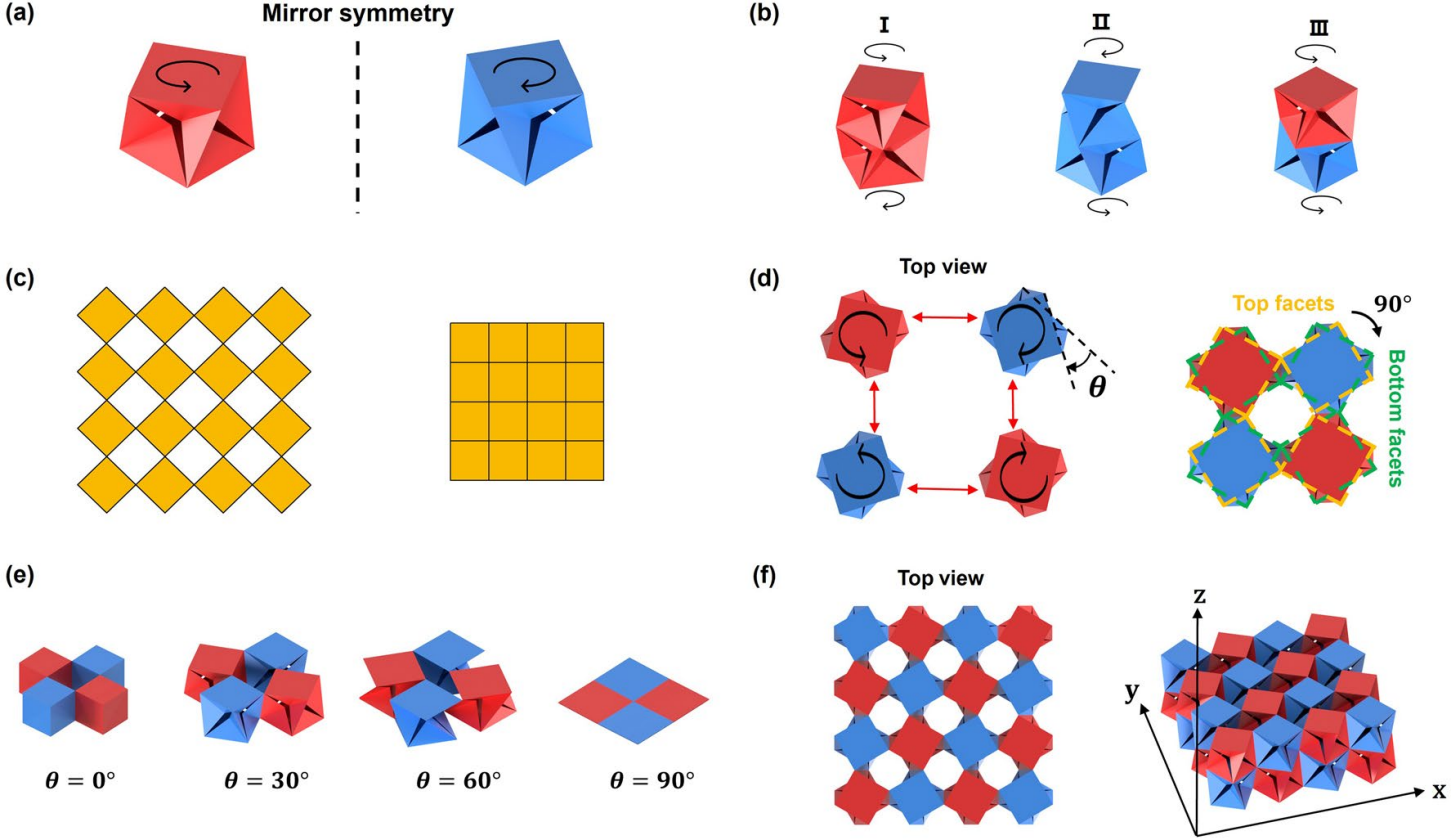
Multi-unit FP-ori structures. (**a**) Two basic chiral FP-ori units. (**b**) Two-layer stacked FP-ori structures. (**c**) Deployed and compacted configurations of square kirigami tessellation. (**d**) Basic unit of FP-ori tessellation structure. (**e**) Folding process of a single basic unit. (**f**) Two-layer FP-ori tessellation structure.

When all the parameters of two FP-ori units are the same but mirror-symmetrical under the given folding angle, the only difference between these two FP-ori units is the rotation direction between the top and bottom facets, which is referred to as chirality. Two symmetrical cubic FP-ori units are depicted in Fig. 5a. The rotation direction of the left (red) one is counterclockwise and that of the right (blue) one is clockwise. By combining these two FP-ori units, three types of two-layer stacked FP-ori structure can be created, as shown in Fig. 5b. If two FP-ori units with the same rotation direction are combined, the rotation direction of the stacked structure remains unchanged. When a clockwise FP-ori unit is combined with a counterclockwise one, the top and bottom facets can be regarded as relatively static with only the middle part rotating, which is similar to Kresling origami. By stacking FP-ori units with different mechanical properties, complex mechanical properties, such as multi-stability, can be achieved (Please see Supplementary Material, section 4 for details). Therefore, the rotation directions and mechanical properties of vertically stacked FP-ori are both programmable, making them applicable in the design of deployable structures such as crawling robots^35^ and robotic arms^36^.

### Planar tessellation configuration

FP-ori can also be combined into a planar tessellation configuration, drawing inspiration from square kirigami tessellation. In Fig. 5c, compacted and deployed configuration of such kirigami with periodic square tiling is exhibited. The cuts along the edges of the squares are designed to allow for rotational in-plane deployment around a set of hinges^37^. A basic unit of the tessellation structure is formed by connecting four pairwise chiral FP-ori units, as shown in Fig. 5d. The yellow and green dashed lines represent the contour of the top and bottom facets, respectively. The FP-ori tessellation structure can be regarded as composed of a square kirigami tessellation and another one rotated by $90^\circ$. Figure 5e illustrates the folding process of the tessellation structure when all bottom facets are fixed on the same plane. Using this method, the multi-unit FP-ori structure can be extended in three directions ($x$, $y$, and $z$), as shown in Fig. 5f. Similar to kirigami tessellation, the FP-ori tessellation structure exhibits a negative in-plane Poisson’s ratio. Additionally, owing to the properties of FP-ori itself, the structure also has a negative out-of-plane Poisson’s ratio (Please see Supplementary Material, Sect.  5 for details on the negative Poisson’s ratio calculation). In comparison to planar kirigami tessellation, 3D FP-ori tessellation offers one additional designable dimension. Consequently, FP-ori tessellation is a novel structure with a negative Poisson’s ratio that holds great potential for broad application in the field of functional metamaterials^38^.

## Discussion

We proposed FP-ori, a rigid-foldable origami inspired by Kresling origami, and conducted a comprehensive analysis of its geometry and mechanical behaviors using the geometric method, FEM simulation, energy model and experiment. To achieve rigid-foldability, FP-ori incorporates several cut lines to Kresling origami. We calculated its geometric relationships between parameters and verified them using 3D modeling software. Different parameter values for FP-ori were found to yield various behaviors, including self-locking, flat-foldability, and bistability. The sole condition for achieving rigid and flat-foldability is $\alpha =45^\circ$, which also serves as the boundary between the two different self-locking types. In addition, for the second type of self-locking ($\Sigma 3$-$\Sigma 4$ self-locking), the existence of bistability depends on the geometric parameter values of FP-ori. FEM simulation was applied to verify the tunable mechanical behaviors of FP-ori with different parameter values and the results aligned with energy model analytical result and experiment. Furthermore, we proposed two methods for constructing multi-unit FP-ori structures. The first method leverages chirality to control the rotation direction of the multi-unit stacked FP-ori structure. The second method was inspired from kirigami tessellation, combining multiple FP-ori units to create a multi-layer tessellation structure with a significantly negative Poisson’s ratio.

In addition, we noticed that Yasuda proposed a truss structure inspired by Kresling origami^39^, which possesses similar tunable mechanical behaviors with FP-ori. Compared with it, FP-ori exhibits several advantages: First, the FP-ori modified method is not limited to truss structures, but can be applied to origami structures, which has potential in deployable structures that prohibit removing facets. Secondly, FP-ori with $\alpha =45^\circ$ has both rigid-foldablity and flat-foldability, while Yasuda’s truss model can only fold a distance equivalent to 15% of its initial height in zero-stiffness mode. Thirdly, FP-ori units can be vertically and horizontally connected, providing additional designable dimensions than other modified Kresling origami structure.

In summary, FP-ori with $\alpha =45^\circ$ can achieve both rigid and flat-foldable, resulting in a considerable variation in volume, indicating this type of FP-ori is well-suited for deployable structures and origami robots. Moreover, FP-ori structures with other parameter values have potential as origami metamaterials because of their tunable mechanical behavior. However, in the process of applying origami theory to practice, developability is a crucial issue that cannot be overlooked. Therefore, for rigid origami with thickness, preserving the properties of zero-thickness origami within it will be a significant focus of our future research. Increasing the freedom of creases appropriately may be an effective approach to addressing interference issues caused by thickness. Additionally, combining origami structures with magnetic materials, shape-memory materials, and thermo-responsive materials to create self-folding origami structure^40–42^ is another research direction for us.

## Methods

The thickness of the paper used in the article was 0.35 mm and the fabrication process followed Movie S1.

To design the 3D models of FP-ori in Figs. 2c, 3b, and 5, we initially created a zero-thickness model for each facet using Autodesk Inventor Professional 2021 and connected them along the creases. The facets were capable of rotating along a crease but cannot be translated or deformed, which satisfies the origami theory. To control the configuration of FP-ori, we ensured that the central axes of the top and bottom facets were coincident and set an angle between two adjacent facets.

For the FEM simulation results presented in Fig. 4b and c, we employed a static, general step to analyze the compression process and utilized a truss model to simulate the mechanical behaviors of FP-ori. Each truss consisted of only one element, ensuring that it only had axial stress. During the compression, we applied a displacement along the height direction to the vertices of the top facet. The strain energy curve was used to represent the change in deformation energy throughout the folding process.

### Supplementary Information

Supplementary Movie S1.

Supplementary Movie S2.

Supplementary Movie S3.

Supplementary Information.

## Data Availability

The data that support the findings of this study are available from the authors on reasonable request. The authors declare that the data supporting this study’s findings are available within the article and the corresponding supplementary information files.
