# Supplementary material for: Rigid-foldable cylindrical origami with tunable mechanical behaviors
Source: Sci Rep. 2024 Jan 2;14:145. doi: 10.1038/s41598-023-50353-4 (PMC10762141; doi:10.1038/s41598-023-50353-4)
Supplement: Supplementary file 4 — Supplementary Information. [file 41598_2023_50353_MOESM4_ESM.pdf]

Supplementary Materials

Rigid-Foldable Cylindrical Origami with Tunable Mechanical Behaviors

Fengrui Liu[1], Tatsuro Terakawa[1,*], Siying Long[1], and Masaharu Komori[1]

[1] Department of Mechanical Engineering and Science, Kyoto University, Kyoto daigaku-katsura, Nishikyo-ku, Kyoto 615-8540, Japan

[*]Corresponding author: terakawa@me.kyoto-u.ac.jp

1. Crease patterns of triangular, pentagonal, and hexagonal foldable prism origami

In Fig. 1 (main article), we showed that the FP-ori method can be applied to all regular prisms to achieve rigid folding. The crease patterns, deployed configurations, and flat-folded configurations of a triangular prism with $\alpha = 30°$, a pentagonal prism with $\alpha = 54°$, and a hexagonal prism with $\alpha = 60°$ are shown in Fig. S1. These three examples all satisfy the flat-foldability conditions obtained in the main article, so the flat-folded configuration can be obtained without considering the intermediate process of the bistable state.

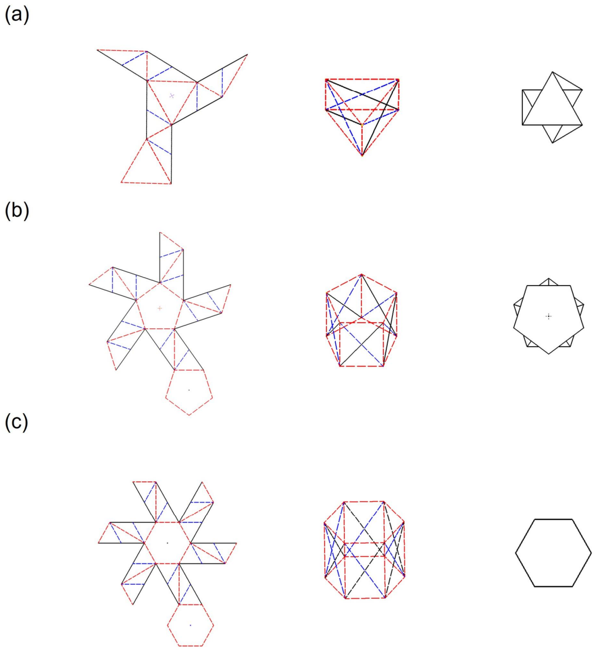

Figure S1. Crease patterns, deployed configurations and flat-folded configurations of various FP-ori structures. (a) Triangular FP-ori with $\alpha = 30°$. (b) Pentagonal FP-ori with $\alpha = 54°$. (c) Hexagonal FP-ori with $\alpha = 60°$.

2. Folding process by FEM simulation

Figure S2 illustrates the stress variation during the folding process, with colors indicating the magnitude of the axial stress. Consequently, changes in color reflect alterations in stress throughout the folding process. When $\alpha = 30°$ or $54°$, the color of the trusses gradually becomes darker as the folding process advances and the stress does not become zero even when the structure is completely flattened. However, when $\alpha = 45°$, the stress remains nearly zero throughout the process, verifying the rigid foldability of FP-ori. For $\alpha = 60°$, the figure shows the stress at zero in both the initial and flattened configurations, with stress appearing in the intermediate states. The initial increase and subsequent decrease of the stress in the truss are consistent with bistability.

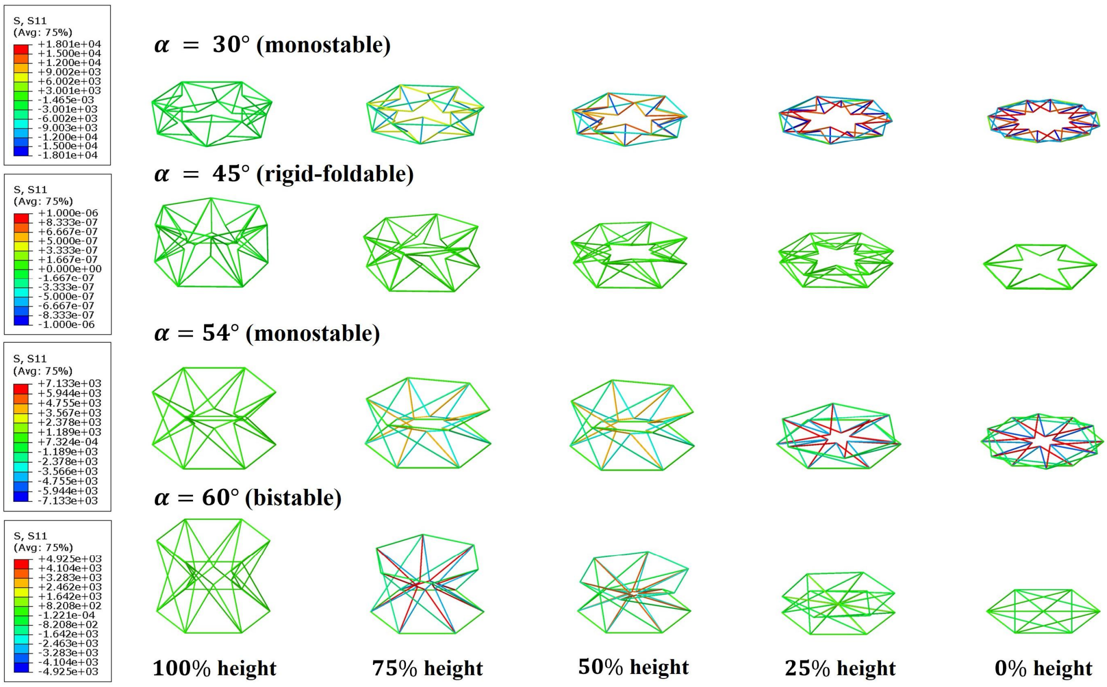

Figure S2. Stress variation during the folding process for various α values.

3. Energy Model of the FP-ori truss structure

To evaluate how the energy changes with the compression, we have developed an energy model for FP-ori based on a truss model. Because the boundary of monostable and bistable states are within the Σ3 and Σ4 self-locking, the configuration is still considered as the initial configuration, consistent with the FEM simulation. In this truss energy model, the trusses comprising the top and bottom facets are considered rigid, while the axial trusses between the top and bottom facets are treated as elastic trusses with a modulus of elasticity E. Figure S3 displays a schematic and a top view of the truss model, with red (CE and EB), blue (AE and ED), and green (CB) representing trusses with different deformations. Next, we calculated the elastic strain energy U of the FP-ori structure. We defined u as the compression distance, h_0 as the initial height of the FP-ori, with a_{1_0}, a_{2_0}, and b_0

representing the initial lengths of the three different kinds of axial trusses, θ_0 as the rotation angle of the top and bottom facets at self-locking, θ as the additional rotation angle generated during the compression process, R as the radius of the circumscribed circle of the top and bottom facets, and r as the radius of the inscribed circle composed of points E in each limb. Based on geometric relationships, we can derive the length changes of the three types of trusses during the compression process as follows:

$$a_1 = \sqrt{(h_0 - u)^2/4 + R^2 + r^2 - 2Rr\cos(\theta_0/2 + \theta/2)} \tag{S1}$$

$$a_2 = \sqrt{(h_0 - u)^2/4 + R^2 + r^2 - 2Rr\cos(\theta_0/2 - \theta/2)} \tag{S2}$$

$$b = \sqrt{(h_0 - u)^2 + 4R^2(\sin(\theta_0/2 + \theta/2))^2} \tag{S3}$$

Based on Eq. (S1), (S2), (S3), the total elastic energy can be expressed as

$$U(u,\theta,r) = n\text{EA}\ (b_0{\varepsilon_b}^2 + 2a_{1_0}{\varepsilon_{a_1}}^2 + 2a_{2_0}{\varepsilon_{a_2}}^2)/2 \tag{S4}$$

where ε_{a_1}, ε_{a_2}, ε_b are the stains of the trusses and n represents the number of sides of the polygon. Applying the principle of minimum potential energy to Eq. (S4), the following two partial differential equations can be obtained:

$$\partial U/\partial\theta = 0 \tag{S5}$$

$$\partial U/\partial r = 0 \tag{S6}$$

To obtain the solution to the system of equations, MATLAB is utilized to establish the relationship between the compression distance and the model's deformation energy. When incorporating the material parameters as well as the cross-sectional area of the trusses set in Abaqus, it becomes evident that the analytical results of the truss energy model closely match the results of FEM simulations shown in Fig. 4(b).

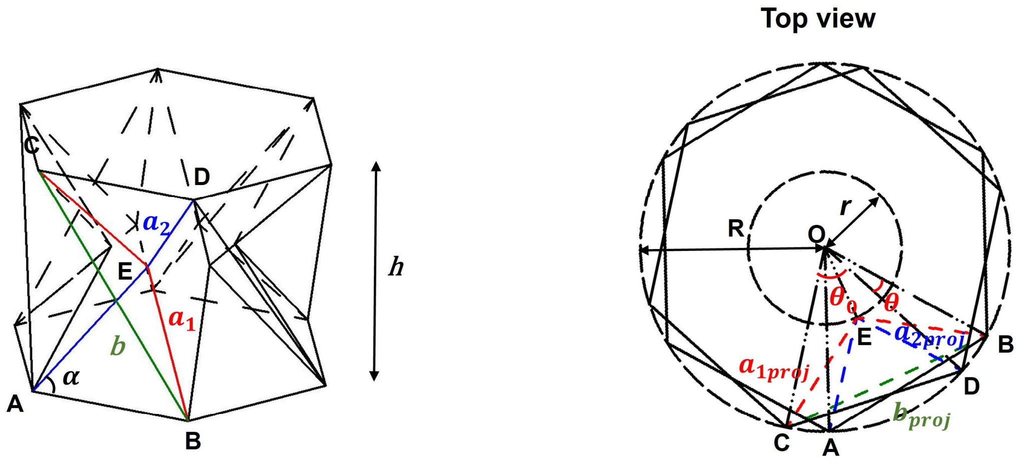

Figure S3. Schematic diagram and top view of FP-ori truss model with $n = 6$ and $\alpha = 60°$.

4. Multi-stable structure

To show the properties of the stacked FP-ori structure, a simulation was conducted. Here, we focus on the type-II stacked FP-ori shown in Fig. 5(b) (main article), which is composed of two hexagonal FP-ori units with $\alpha = 60°$. The section areas of the truss were set to 1 and 0.5 mm^2, respectively, in the simulation to distinguish the lower and upper FP-ori units. Other parameters were the same as those in the main article. Figure S4 shows the variation of strain energy during the folding process. It shows five configurations of the folding process (A, B, C, D, and E). The strain energy in configurations A, C, and E is zero, where FP-ori is at a steady state. In configurations B and D, the strain energy takes maximum values. The stress variation corresponding to these five configurations is also shown in the figure. The stress in the initial state (configuration A) is zero. Then, the upper layer with a smaller section area is flattened first (configuration C). The lower layer then begins to be compressed until the upper layer is flattened (configuration E). The variations of strain energy and stress verify that multi-stability can be obtained when FP-ori units are stacked. FP-ori with $n = 6$ and $\alpha = 60°$ was shown to be bistable in the main article. Therefore, there may be three or more steady states if two or more such FP-ori units are combined.

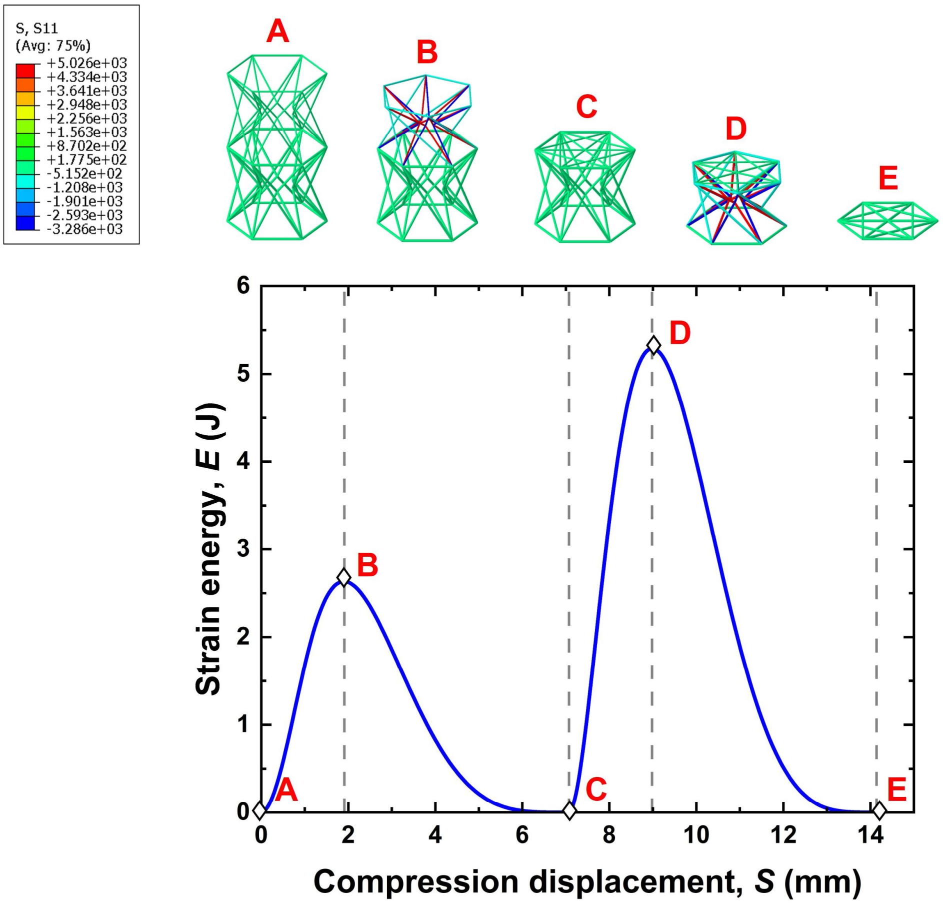

Figure S4. Strain energy and stress variation of stacked FP-ori structure composed of two FP-ori units with $n = 6$ and $\alpha = 60°$.

5. Calculation of negative Poisson's ratio of tessellation structure

The FP-ori unit in the upper-left corner on the left side of Fig. S5 is used as an example to calculate the negative Poisson's ratio of the FP-ori tessellation structure. The vertices of the bottom and top facets are marked as ABCD and EFGH, respectively. From the top view, CG is parallel to AE, so for a unit, the width *w* can be considered to be the projection distance from point G to point A on the bottom facet ABCD. Point O is the center of the top facet, $\theta \in [0,\pi/2]$ is the rotation angle between the top and bottom facets, and *l* is the side length of the square. Therefore, the distance between A and G can be calculated as

$$w = \sqrt{2}l\cos\gamma = \sqrt{2}l\cos(\theta/2) \quad \text{(S1)}$$

Similarly, *s* can be calculated as

$$s = w \quad \text{(S2)}$$

Substituting $\alpha = 45°$ and $\beta = 90°$ into equation (6) (main article) yields

$$h = l \cdot \sqrt{\cos\theta} \quad \text{(S3)}$$

Therefore, the Poisson's ratios of one unit can be calculated as

$$\nu_{sw} = -\varepsilon_s/\varepsilon_w = -1 \quad \text{(S4)}$$

$$\nu_{hw} = \nu_{hs} = -\varepsilon_h/\varepsilon_w = -\frac{\sqrt{2}\cos(\theta/2)}{\sqrt{\cos\theta}} \quad \text{(S5)}$$

For the tessellation structure, *m*, *n*, and *p* represent the number of FP-ori units in the directions of *x*, *y*, and *z*, respectively. The following equations can be obtained:

$$S = ms \quad \text{(S6)}$$

$$W = nw \quad \text{(S7)}$$

$$H = ph \quad \text{(S8)}$$

Therefore,

$$\nu_{SW} = -\varepsilon_S/\varepsilon_W = -m/n \quad \text{(S9)}$$

$$\nu_{HW} = (p/n)\,\nu_{hw} \quad \text{(S10)}$$

$$\nu_{HS} = (p/m)\,\nu_{hs} \quad \text{(S11)}$$

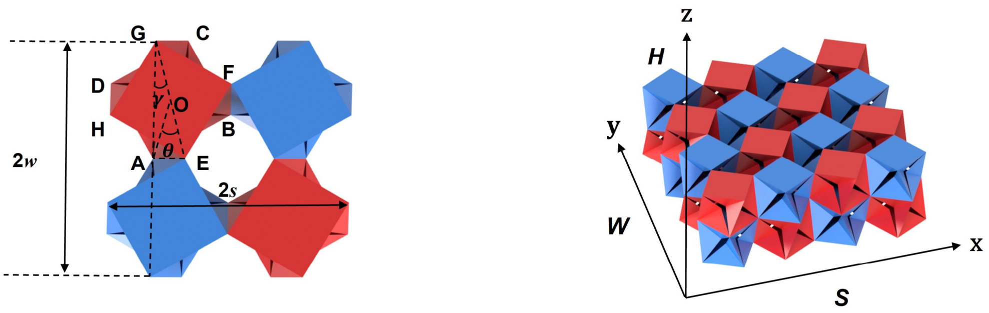

Figure S5. Schematic diagram of tessellation structure.
